# Implementation of Sexual and Reproductive Health Education Policy in Schools in Asia and Africa: A Scoping Review

**DOI:** 10.1080/19317611.2024.2409129

**Published:** 2024-10-08

**Authors:** Rhea Khosla, Victoria Tzortziou Brown

**Affiliations:** a Medical School, University of Birmingham, Birmingham, UK; b Wolfson Institute of Population Health, Queen Mary’s University of London, London, UK

**Keywords:** SRH education, policy, implementation, Asia, Africa

## Abstract

**Objectives:**

This review aimed to map and synthesize existing literature on sex and reproductive education policy implementation in primary and secondary schools in Asia and Africa.

**Methods:**

Database searches yielded 24 relevant articles, which underwent thematic analysis.

**Results:**

Most studies were conducted in Africa and looked at barriers to implementation. Studies assessing implementation showed it was incomprehensive. Barriers were: policy/curriculum issues, societal opinions, teaching discomfort, lack of educator training, and lack of sufficient economical support.

**Conclusions:**

Based on the limited evidence, a cultural shift to reduce stigma seems necessary, alongside teacher and student involvement in policy formulation and implementation monitoring.

## Introduction

The sexual and reproductive health (SRH) of adolescents has been somewhat neglected since the millennium. When the Millennium Development Goals were originally introduced, SRH was omitted. After years of advocacy, a reproductive health target was then included in the official list of goals in 2008, after the “Millennium plus 5” Summit in 2005 (Hulme, 2009). In 2015, this was further revised in the “2030 Agenda for Sustainable Development” led by the United Nations, which included a target aiming for universal access to SRH services by 2030 (WHO, 2022). Whilst there has been global progress in outcomes for SRH (Starrs et al., 2018), adolescents remain a particular group at risk.

SRH education, a term used in this paper to encompass sexuality, sex, sexual health, and reproductive education, provides an outlet to inform these young people about the importance of their sexual health, alongside their human, including sexual, rights. Despite acknowledging its importance, SRH education remains of low political priority in Asia and Africa (Clarke, 2010; Onono et al., 2019). Policy mandating such education is essential to promote SRH, especially in primary and secondary schools which are a good environment to regulate and maximize delivery. However, whilst there are policies in place in Asia and Africa (UNFPA et al., 2020; Wangamati, 2023), it is unclear whether these policies are implemented. In 2012, UNESCO reported that only a small number of adolescents in most low/middle-income countries were reached by SRH education programs (UNESCO, 2014), urging for assessment of current SRH education policy.

This scoping review aimed to answer two key questions concerning SRH policy implementation:

1. To what extent are SRH education policies implemented in Asia and Africa?
2. What are the barriers to policy implementation?

## Methods

**Figure 1.**
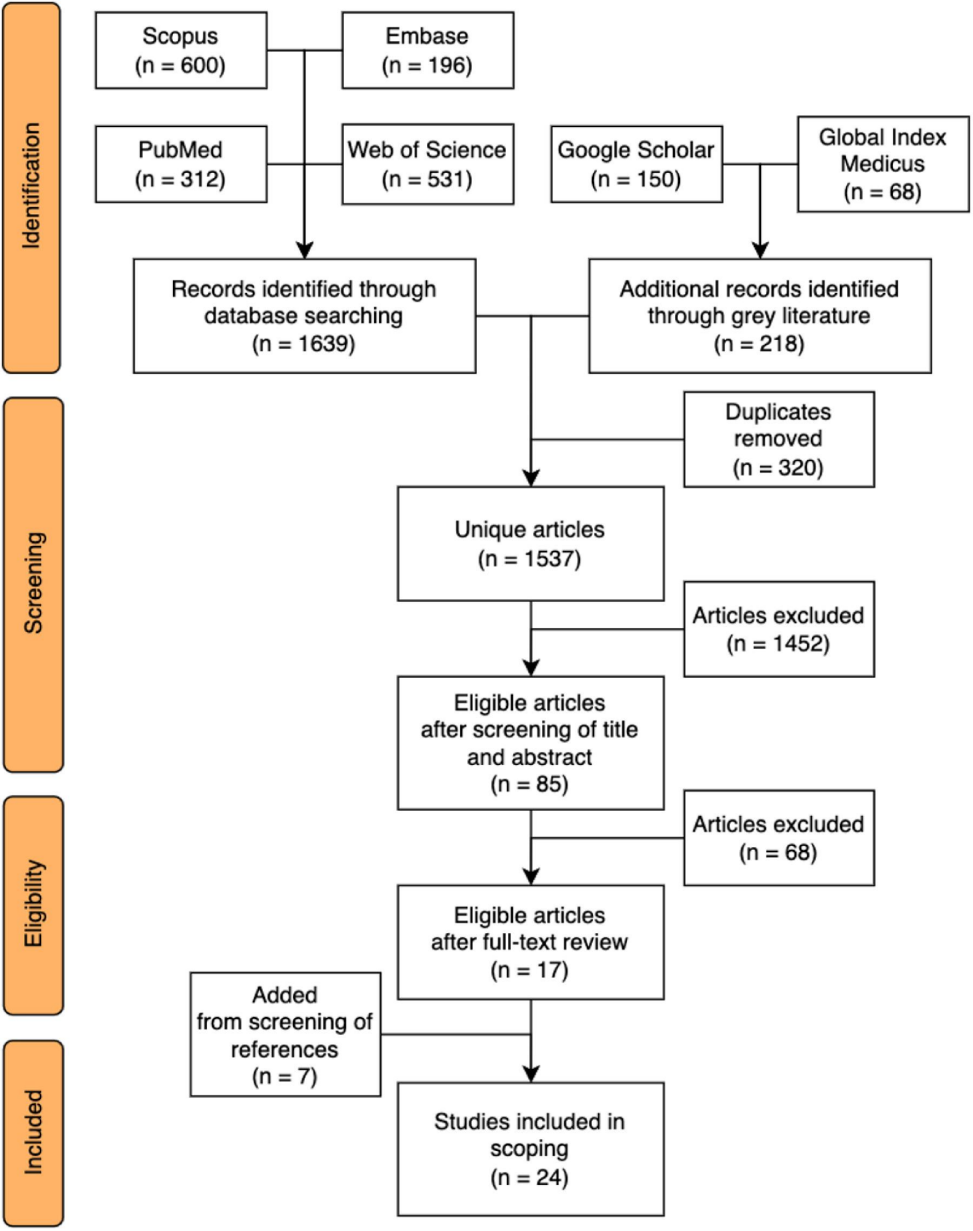
Flowchart of included articles.

The updated methodological guidance for scoping reviews by Peters et al. (Peters et al., 2020) was used. The database search is outlined in Figure 1. A total of 1639 articles were retrieved from four relevant databases (PubMed, Scopus, Embase, Web of Science) in February 2023. Google Scholar and Global Index Medicus were additionally searched for grey literature. The grey literature search returned 218 articles.

The keywords for the searches are outlined in Appendix A, with the specific database searches listed in Appendix B.

All citations were exported into Zotero, and duplicates were removed, returning 1537 unique articles. 17 studies remained after the screening of unique articles (titles, abstracts, and full texts). An additional seven papers were retrieved by reviewing references of selected articles. Articles identified by the search process underwent thematic analysis using the Braun and Clarke (2006) framework (Braun & Clarke, 2006).

### Inclusion and exclusion criteria

Articles on the implementation of SRH education policy in primary and secondary schools in Asian and African countries were included. The inclusion and exclusion criteria (Table 1) were developed using the PCC tool, as described in Appendix C.

**Table 1.** Inclusion and exclusion criteria.

Inclusion criteria	Exclusion criteria
From Asia/Africa	Policy recommendations without considering existing policy
In English	No explicit consideration of SRH education policy/curriculum
Criticize SRH education policy implementation	Not school-based
Policies/curricula criticized were nationally implemented	Published before 2000
School-based	Focus not on Sexuality Education
Full text available	Policy recommendations without considering existing policy

## Results

### Included studies

The 24 included studies and their characteristics are listed in Appendix D.

**Figure 2.**
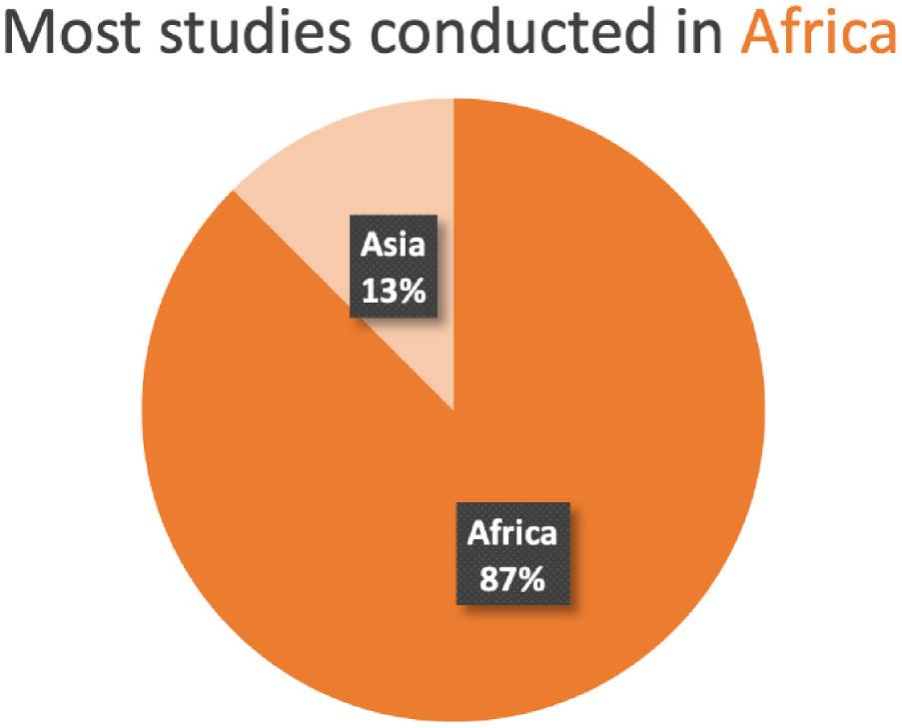
The proportion of studies from each continent.

**Figure 3.**
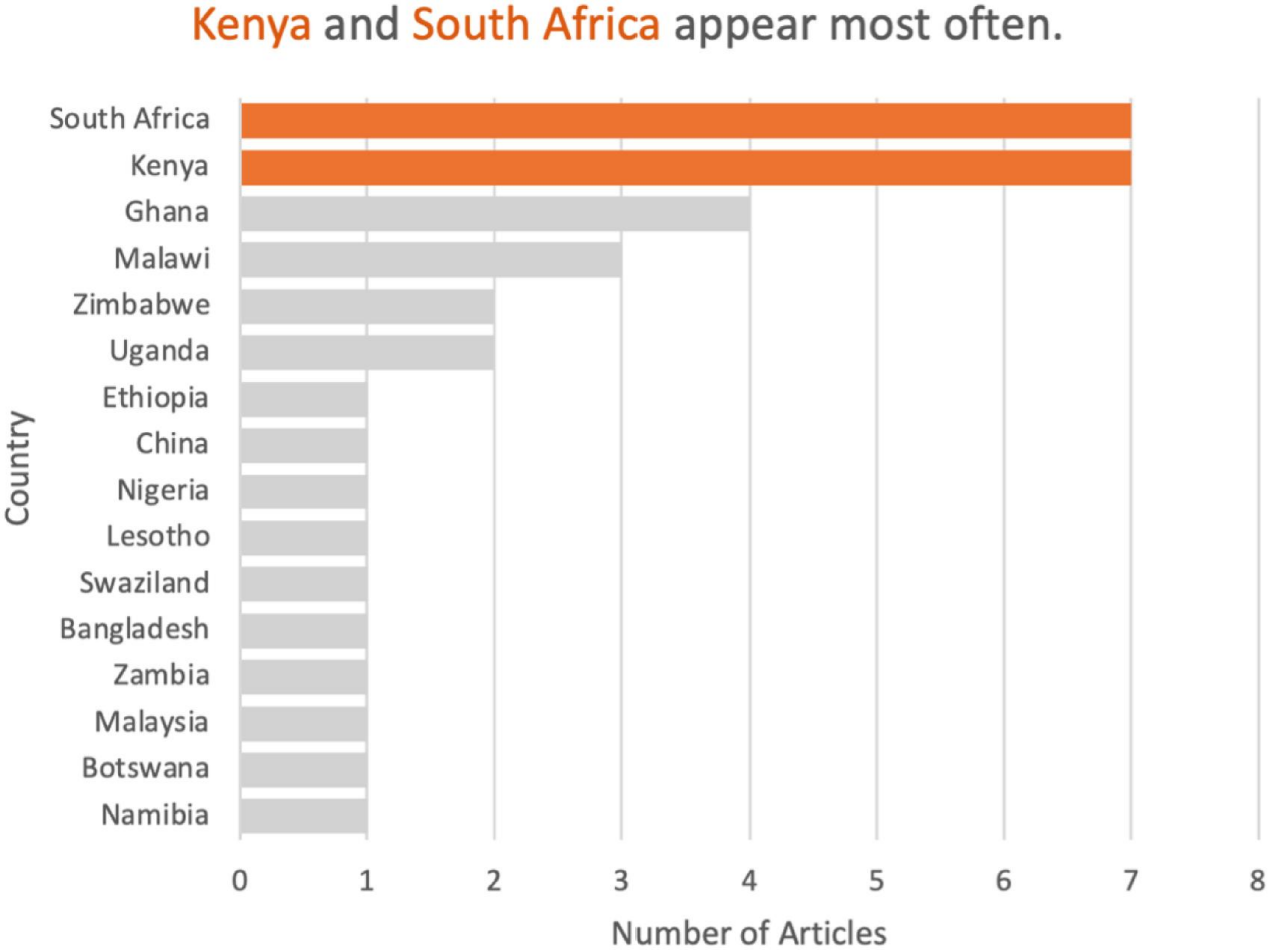
Bar chart of number of articles per country (N.B. some studies included more than one country).

**Figure 4.**
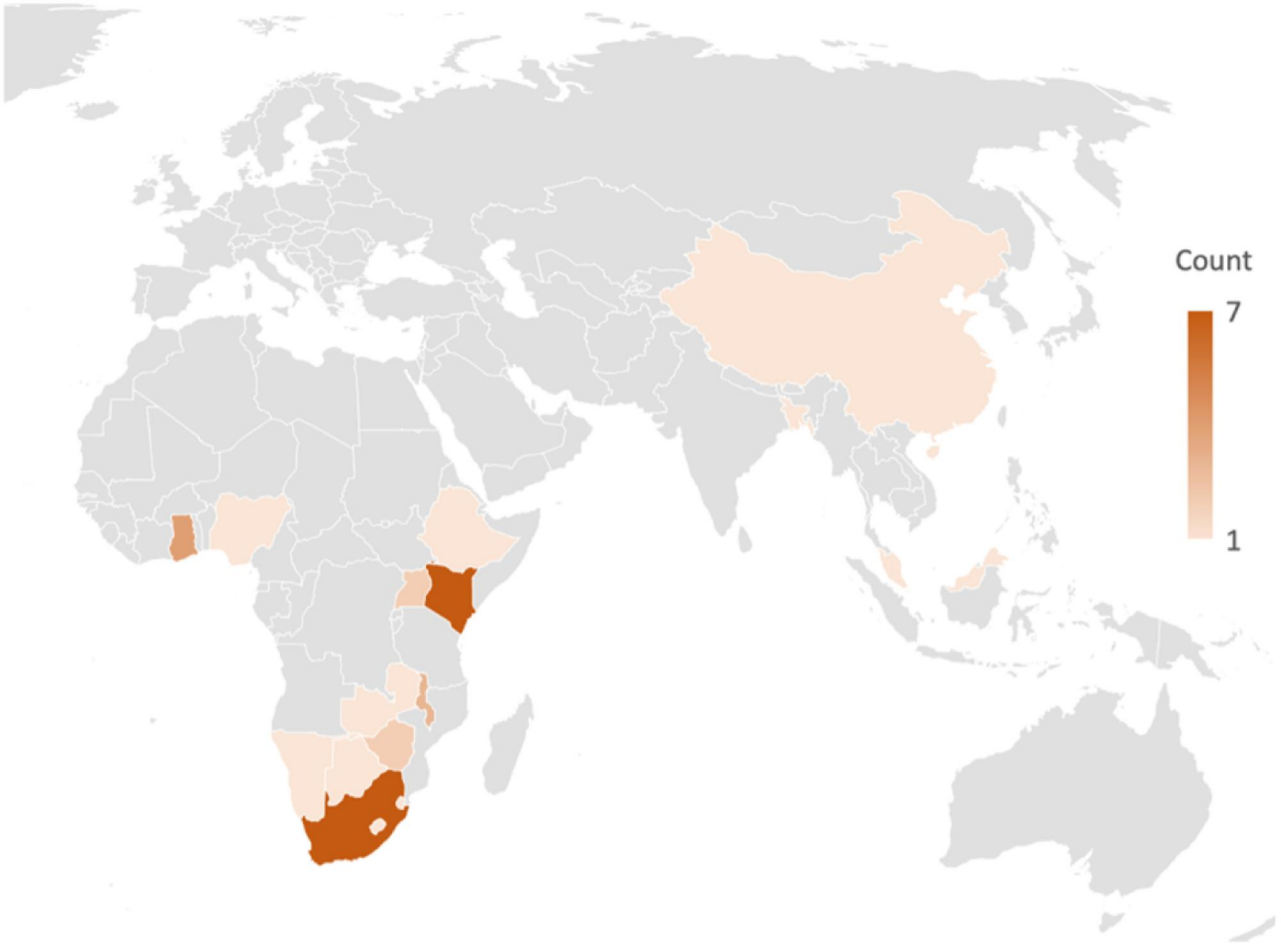
Map of included studies by country (N.B. some studies included more than one country).

Of the 24 studies, 21 studies were conducted in Africa (Figure 2), with the largest proportion conducted in Kenya and South Africa (7 each). For most countries in Asia and Africa, no studies were conducted (Figures 3 and 4).

Of the 24 studies, 15 studies looked into the degree and scale of SRE policy implementation in 8 different countries. All studies included (24/24) looked into the barriers to implementation.

Of the 24 studies, 8 used mixed methods and the rest were mostly qualitative. Qualitative data collection methods mainly included questionnaires and semi-structured interviews, involving participants such as students, educators, and key informants such as policymakers, parents, and religious leaders. Most studies conducted primary research, while five studies utilized secondary data. Two articles reviewed both primary and secondary data, one solely reviewed secondary data, and two analyzed secondary data. Additionally, five papers examined policy documents to identify barriers to implementation.

A total of 14 qualitative studies and 10 mixed studies were analyzed.

### Studies on the degree of SRE policy implementation

SRE policies were implemented to different degrees in different countries. Key themes on the degree of implementation were: insufficient hours dedicated to SRH education, lack of integrity in implementation and age at the start of SRH education programme.

#### Insufficient hours

Whilst policies tended to outline how much time should be dedicated to SRH education, several studies reported that these guidelines were not followed.

One study found that Chinese schools allocated insufficient time to SRH education (as per policy), with significant and unexplained variations in time allotted to the subject. For example, Grades 7 and 8 were only allocated 1–8 hours, and even less time was devoted to Grade 9 (UNESCO, 2018).

Insufficient time was also dedicated to SRH education in Ghana according to a study. National policy requires schools to allocate 16–21 hours for SRH education each term, though many schools fell short of this. According to teachers, under one-third of schools dedicated more than 10 hours to the subject (Awusabo-Asare et al., 2017).

Additionally, in Kenya, specifically in Homa Bay and Mombasa, over 85% of schools taught SRH education for at least 6 hours per term in Form 2 and for 6 or more hours in Form 3. However, in Nairobi, less than 40% of schools did the same for Form 2 and only 65% did so for Form 3 (Sidze et al., 2017).

#### Integrity and degree of implementation

Issues were identified with the integrity of implementation, such as the lack of correct implementation of policy by teachers. For example, while 99% of Kenyan schools reported policy implementation, teachers were not following the HIV/AIDS curriculum (Ndambuki et al., 2006). The questionable integrity of implementation was deduced from questionnaire responses, where participants referred to a different health program instead of the HIV/AIDS policy. 95% of teachers interviewed were aware the policy existed, but not of its content, so could not be implementing it (Wittenberg et al., 2007). In Ghana, only 8% of learners felt all necessary topics were covered, despite 83% of teachers claiming to do so (Awusabo-Asare et al., 2017).

The level of implementation of policy by schools was variable. In Malawi, only one-third of adolescents reported their schools offered SRH education, and attendance was low even where it was offered (Wittenberg et al., 2007). In Nigeria, whilst SRH education was mandated at both primary and secondary schools, a Ministry of Education official estimated only 10% of schools covered this topic (Kunnuji et al., 2017). In South Africa, 70% of teachers reported teaching HIV/AIDS education (Mathews et al., 2006). However, few schools had clear initiatives to implement the HIV/AIDS curriculum. A quarter of schools surveyed were not implementing the policy, and 42% admitted implementation issues (Raniga, 2014).

There was some good level of implementation shown too. In one study conducted in Ghana, Kenya, Guatemala, and Peru, Ghana scored highest for overall implementation quality (Keogh et al., 2019). In Ghana, SRH education was incorporated into both curricular and extracurricular^1^ activities. The school environment for SRH education was overall best in Kenya, although the curriculum itself^2^ scored lowest in the surveyed countries (Keogh et al., 2019).

#### Age at implementation

SRH education was implemented fairly early in most countries. In Ghana, interviews with teachers and the Municipal Health Education Coordinator confirmed policy implementation in schools for all ages (Ocran et al., 2022). By the end of primary school, over 75% of Ghanaian students had been exposed to SRH education, and by the end of junior high school, 97% had been exposed. Similarly, 96% of Kenyan students were first exposed to SRH education in primary school (Sidze et al., 2017).

However, in Malawi, a different trend was seen. Students of most ages had not received any SRH education; a study found under 20% of 12- to 14-year-olds, and only 14% of females and 31% of males aged 15–19 had received SRH education (United Nations Population Fund, 2012).

#### Implementation data summary

Table 2 summarizes the key quantitative data.

**Table 2.** Policy implementation data summary.

Country	Data on SRH education	Year
China	1–8 hours per semester for grades 1–8 (UNESCO, 2018)	2018
Ghana	83% teachers teaching all topics (Awusabo-Asare et al., 2017)	2017
	8% students learning all topics (Awusabo-Asare et al., 2017)	
	31% schools >10hrs per term in form 2 (Awusabo-Asare et al., 2017)	
	25% schools >10hrs per term in form 3 (Awusabo-Asare et al., 2017)	
Kenya	50.7% teacher implementing (Ngarari, 2010) 79.1% students taught (Ngarari, 2010)	2010
	86%/95%/39% schools implement >6hrs per term in form 2 (Mombasa/Homa Bay/Nairobi) (Sidze et al., 2017)	2017
	92%/88%/65% schools implement >6hrs per term in form 3 (Mombasa/Homa Bay/Nairobi) (Sidze et al., 2017)	
Malawi	13%:19% students taught (12–14 years, F:M) (Wittenberg et al., 2007)	2007
	14%:31% students taught (15–19 years, F:M) (Wittenberg et al., 2007)	
	33%:39% (student reported schools F:M)	
Nigeria	10% schools implementing (estimate) (Kunnuji et al., 2017)	2017
South Africa	70% teachers implementing (Mathews et al., 2006)	2006
	74% schools implementing (Raniga, 2014)	2014
	70% included STI teaching (Ramalepa et al., 2022)	2022
Swaziland	51% schools implementing (United Nations Population Fund, 2012)	2012

### Barriers to implementation

Six key themes were found through thematic analysis. These were: Policy issues, Issues with curriculum content, Societal opinions and stigma, Discomfort in teaching, Lack of educator training, and Lack of resources.

#### Policy-related limitations

Although SRH education has been shown to be an effective tool in reducing stigma (Hong et al., 2022), several countries’ policies were limited by laws criminalizing homosexuality (United Nations Population Fund, 2012). Moreover, even whilst emphasizing students’ right to access accurate information on SRH, policy restrictions limited curriculum content, with cultural and religious beliefs further restricting this scope (Obare & Birungi, 2013).

The included studies highlighted that inadequate guidelines and strategies were provided for interpreting policies (Koza & Mushoriwa, 2018; Ndambuki et al., 2006; Ngarari, 2010; Sidze et al., 2017). Teachers were expected to create their own SRH education syllabus, without policy documents or guidelines. In addition, policies were sometimes in the form of guidance rather than being mandatory (Mugweni et al., 2014; Ngarari, 2010), leaving teachers to decide whether to implement them at their discretion. Policies sometimes lacked focus and did not include sufficient strategies for SRH education (Ramalepa et al., 2022). In Malawi, SRH education policy only applied to public, and not private, schools, revealing a lack of comprehensive coverage (Wittenberg et al., 2007). In South Africa, policy failed to account for socio-cultural and socioeconomic differences in rural schools (Raniga, 2014).

SRH education policies often lacked input from key stakeholders, including students and teachers, meaning policies did not reflect the reality of students’ SRH needs (Keogh et al., 2018; Koza & Mushoriwa, 2018; Ndambuki et al., 2006; Ngarari, 2010; Obare & Birungi, 2013; Ocran et al., 2022). Such limited engagement sometimes meant that policymakers’ personal opinions could negatively impact policy content, such as the exclusion of contraception due to concerns about promoting sexual behavior (Sidze et al., 2017). This was observed, for example, in Nigeria, where political stakeholders threatened policy implementation, causing delays in implementation (Kunnuji et al., 2017). Similarly, in Ghana, a government official expressed a desire to maintain a curriculum within traditional norms (Awusabo-Asare et al., 2017). Where consulted, student perspectives were not always taken seriously and were not utilized in curriculum formulation (Keogh et al., 2018). Additionally, some felt that better stakeholder involvement in policymaking would improve understanding of policy (Ninsiima et al., 2020).

Nonetheless, such stakeholder engagement was not always supportive. In Nigeria, between 2011 and 2012, the Ministry of Education conducted policy workshops within the community. There were requests for the removal of curriculum items deemed “culturally inappropriate” which resulted in concerns that such a move would reduce policy quality (Kunnuji et al., 2017).

Implementation was not regularly monitored in many countries (Keogh et al., 2018; Kunnuji et al., 2017; Ngarari, 2010; Ninsiima et al., 2020; Raniga, 2014; Sidze et al., 2017), partly due to lack of clarity on the responsibility for monitoring, with individuals holding either the government, schools, or both accountable (Keogh et al., 2018). For example, in Ghana, 30% of headteachers had never evaluated teacher delivery on SRH education, despite conducting general teacher evaluations (Awusabo-Asare et al., 2017). Similarly, in Kenya, 39% of schools did not evaluate teacher delivery of SRH education (Sidze et al., 2017).

In many countries, education policy was centralized. Centralization can encourage investment in training, improve curriculum development, and promote program standardization (Keogh et al., 2018; Sidze et al., 2017). However, decentralization can allow for contextual adaptation and may reduce delays in implementation (Keogh et al., 2018; Sidze et al., 2017). Irrespective of central or local control, a lack of clarity and accountability sometimes resulted to policy failure. In Uganda, officials were unaware of their designated adolescent SRH committees indicating a lack of committee activity. As a result of insufficient governmental guidance, NGOs carrying out SRH education often lacked regional coordination (Ninsiima et al., 2020) In Kenya, SRH education was decentralized and managed at the district level. However, when districts were visited, there was no acknowldgement of their responsibility for implementation (Ngarari, 2010). Simlarly, policies often lacked references to clear leadership in South Africa, containing contradictory statements on the accountability for implementation in policy documents (Raniga, 2014).

#### Curriculum content limitations

SRH education in several of the countries studied focused on abstinence, preventing effective implementation (Ahmed et al., 2009; Awusabo-Asare et al., 2017; Khalaf et al., 2014; Kunnuji et al., 2017; Ngarari, 2010; Obare & Birungi, 2013; Ocran et al., 2022; Sidze et al., 2017). SRH education in some countries did not cover sufficient contraceptive devices (Awusabo-Asare et al., 2017; Kunnuji et al., 2017; Ngarari, 2010; Ocran et al., 2022), such as in Kenya, where contraceptives were not promoted (Obare & Birungi, 2013).

In some studies, teachers avoided important topics such as relationships, sexual rights, and safe sexual activity, only providing basic knowledge of biology and puberty changes, which did not address the issue of adolescent pregnancies and risky sexual relationships (Awusabo-Asare et al., 2017; Keogh et al., 2019; Khalaf et al., 2014; Ndambuki et al., 2006; Ngarari, 2010; Ninsiima et al., 2020; United Nations Population Fund, 2012).

The focus on abstinence resulted in incomplete information. For instance, where students were told abstinence was the best way to prevent sexually transmitted infections (STIs) (Ngarari, 2010), this did not inform students who were sexually active about their options. In Ghana, 72% of students claimed SRH education taught sex was dangerous for young people, and 82% of teachers taught abstinence was the best or only method of contraception (Awusabo-Asare et al., 2017). Furthermore, SRH education was often presented negatively (Awusabo-Asare et al., 2017; Keogh et al., 2019; Ocran et al., 2022; Sidze et al., 2017). In Kenya, for instance, the teaching of human rights was focused on abuse, rather than teaching students how to respect another person’s bodily autonomy (Sidze et al., 2017).

SRH curriculum content was seen to vary vastly between different schools in the same country (Ahmed et al., 2009; Mugweni et al., 2014; Ngarari, 2010; Wittenberg et al., 2007), due to a lack of direction given on what to teach. In Malawi, South Africa, and Zimbabwe, teachers were free to teach topics without consulting school governing bodies (Ahmed et al., 2009; Mugweni et al., 2014; Wittenberg et al., 2007). This lack of direction sometimes demotivated teachers from implementing the policy (Mugweni et al., 2014). Insufficient government funding also contributed to content inconsistencies, as alternative funders and providers with varying organizational priorities filled funding gaps (Keogh et al., 2018).

Furthermore, SRH education was not treated as a priority and was often integrated into other subjects, rather than being taught as a stand-alone subject (Awusabo-Asare et al., 2017; Keogh et al., 2018; Ndambuki et al., 2006; Ngarari, 2010; Ninsiima et al., 2020; Ocran et al., 2022; Ramalepa et al., 2022; Roy et al., 2022; UNESCO, 2018; Wittenberg et al., 2007) This sometimes resulted in the subject being skipped (Keogh et al., 2018; Le Mat et al., 2021). Instead, teachers and students seemed to give examined subjects more priority (Awusabo-Asare et al., 2017; Keogh et al., 2018; Khalaf et al., 2014; Ngarari, 2010; Sidze et al., 2017; Wittenberg et al., 2007), especially with increased pressures to meet targets for these (Khalaf et al., 2014; Roy et al., 2022).

#### Societal and teachers’ opinions and stigma

SRH education was often viewed negatively in Asia and Africa due to cultural beliefs (Khalaf et al., 2014; Kunnuji et al., 2017; Le Mat et al., 2021). Societal beliefs that SRH education promoted premarital sex prevented implementation (Likupe et al., 2021; Ndambuki et al., 2006) and resulted in some teachers founding it socially unacceptable to discuss certain SRH topics (Ahmed et al., 2009; Awusabo-Asare et al., 2017; Khalaf et al., 2014; Le Mat et al., 2021; Mugweni et al., 2014; Ninsiima et al., 2020; Ocran et al., 2022; Rohleder et al., 2012; Sidze et al., 2017; United Nations Population Fund, 2012; Wittenberg et al., 2007). In Uganda, even politicians and bureaucrats, who feared losing voters’ support, rejected parts of the curriculum that were religiously and culturally sensitive (Ninsiima et al., 2020).

The stigma associated with SRH education often led to demotivation to teach the subject (Le Mat et al., 2021; Mathews et al., 2006; Mugweni et al., 2014; Ninsiima et al., 2020; UNESCO, 2018). Many teachers believed SRH education was not important (Mugweni et al., 2014), especially for students with disabilities (Rohleder et al., 2012), with a lack of support from schools and headteachers (Koza & Mushoriwa, 2018; Mathews et al., 2006; Ngarari, 2010; Sidze et al., 2017). In Kenya, 94.4% of teachers claimed they had insufficient support from other teachers (Mugweni et al., 2014; Ngarari, 2010).

#### Discomfort in teaching

Teachers felt uncomfortable teaching SRH education to students, often due to personal beliefs and embarrassment (Ahmed et al., 2009; Keogh et al., 2019; Koza & Mushoriwa, 2018; Likupe et al., 2021; Mathews et al., 2006; Mugweni et al., 2014; Rohleder et al., 2012; Roy et al., 2022; Sidze et al., 2017; UNESCO, 2018; United Nations Population Fund, 2012): Details such as names of reproductive organs were often omitted (Keogh et al., 2019; Sidze et al., 2017; UNESCO, 2018). In Kenya, 37% of teachers reported embarrassment when teaching SRH education (Sidze et al., 2017; UNESCO, 2018).

Student embarrassment also seemed to prevent engagement with SRH education (Awusabo-Asare et al., 2017; Ninsiima et al., 2020; Ocran et al., 2022; Rohleder et al., 2012; Roy et al., 2022; Sidze et al., 2017). In Ghana and Kenya, 41% and 50% of students respectively cited embarrassment as a reason for not participating, with fears of being shut down by teachers or classmates (Awusabo-Asare et al., 2017; Sidze et al., 2017). In some cases, teachers embarrassed students by addressing them in front of the whole school (Ocran et al., 2022). Some teachers discouraged student questions, replying that they were inappropriate or students would learn such topics when they were older (Roy et al., 2022). Due to class behavior, teachers believed students lacked maturity (Rohleder et al., 2012; Roy et al., 2022).

In several of the studies, some teachers suggested students of different sex should be separated during SRH education due to discomfort and immaturity (Ahmed et al., 2009; Awusabo-Asare et al., 2017; Likupe et al., 2021; Ninsiima et al., 2020; Sidze et al., 2017; United Nations Population Fund, 2012), as well as differences in cognitive and developmental maturity (Ahmed et al., 2009). Male students were asked to leave during teaching about female anatomy and vice versa (United Nations Population Fund, 2012). Contrastingly, according to a study in Malawi, mixed sessions increased student discomfort (Likupe et al., 2021). There was some evidence that students were also embarrassed to be taught by teachers of the opposite sex (Likupe et al., 2021).

#### Lack of educator training

According to the studies, several teachers lacked an understanding of policies and curricula (Awusabo-Asare et al., 2017; Koza & Mushoriwa, 2018; Le Mat et al., 2021; Mugweni et al., 2014; Ocran et al., 2022; Roy et al., 2022; Sidze et al., 2017; United Nations Population Fund, 2012), resulting in poor implementation.

Where implementation training was undertaken, this was brief, non-standardized, and non-comprehensive (Ahmed et al., 2009). 94% of Kenyan teachers felt training was inadequate due to no mandated training and the government’s assumption teachers were already prepared to teach SRH education (Awusabo-Asare et al., 2017; Ngarari, 2010). In Zimbabwe and Nigeria, some teachers had never attended training (Kunnuji et al., 2017; Mugweni et al., 2014):

#### Lack of resources and government support

Teachers often struggled with implementing SRH education due to limited time and resources, especially with large classes of 50–60 students (Ahmed et al., 2009; Awusabo-Asare et al., 2017; Keogh et al., 2019; Khalaf et al., 2014; Kunnuji et al., 2017; Le Mat et al., 2021; Likupe et al., 2021; Mugweni et al., 2014; Ngarari, 2010; Rohleder et al., 2012; Sidze et al., 2017; United Nations Population Fund, 2012). In Kenya, 94.4% of teachers believed resources and time for SRH education were insufficient (Ngarari, 2010). This was due to lack of government funding and support (Awusabo-Asare et al., 2017; Keogh et al., 2018; Khalaf et al., 2014; Kunnuji et al., 2017; Ninsiima et al., 2020; Raniga, 2014; Rohleder et al., 2012). In Malaysia, policymakers believed implementing SRH education policy nationally was unreasonably time-consuming and required significant governmental efforts (Khalaf et al., 2014). Policy centralization compounded this lack of funding; in Ghana, parent-teacher associations raised funds for SRH education, but schools could not accept these until central government’s approval (Keogh et al., 2018).

In South Africa, where resources were provided, they were “too basic” in the form of games (Rohleder et al., 2012). Disparities in resource allocation, especially of essential services including running water, seemed to unfavorably impact all education taught in schools, including SRH (Raniga, 2014).

#### Summary of themes and subthemes

Table 3 summarizes the key themes and subthemes.

**Table 3.** Key themes and subthemes from thematic analysis.

Theme	Subtheme
Policy issues	Policy limitations
	Direct issues with implementation
	Stakeholder involvement
	Insufficient monitoring
	Centralization of policy
Curriculum content	Focus on Abstinence and Biology
	Fear-based teaching
	Lack of standardization, focus, and examination
Societal Opinions and Stigma	Community and Teacher Opinions
	Belief in importance of SRH education
Comfort in Teaching	Teacher comfort
	Student comfort and Maturity
Educator Training	Lack of teacher knowledge
	Lack of training
	Need for specialists
Lack of Resources and Government Support	Money, time, resource allocation

## Discussion

### Summary of findings

The included studies on SRH education policy implementation were limited to only a few countries in Asia and Africa, with the majority focusing on Africa. Most studies collected qualitative data derived from interviews with key stakeholders.

Findings suggested incomplete and inconsistent SRH education policy implementation in most countries, showing insufficient hours dedicated to teaching, education received late into schooling, and discrepancies between teachers, schools, and students on the degree of implementation.

Thematic analysis revealed 6 key themes on the barriers to policy implementation, including policy limitations, curriculum content issues, societal opinions and stigma, discomfort in teaching, lack of educator training, and lack of resources and government support. In addition to preventing implementation, these barriers reduced the impact of SRH education when it was implemented.

### Interpretation of findings and recommendations

#### The need for a cultural shift

The findings suggest the need for a cultural shift in Africa and Asia to overcome the stigma surrounding SRH. This stigma hinders the creation and implementation of effective policies, whilst limiting access to sexual health services for those who need them most (Griffin, 2006).

In cases where religious leaders support SRH education (Ilika et al., 2006), their influence can be used to promote it. In Liberia, religious leaders collaborated with the UNFPA to reduce teen pregnancy rates and pressure the government to improve SRH education policy (Religious Leaders Commit to Sexuality Education in Churches and Mosques in Liberia [Internet], 2017), while in Nigeria, “Action Health Incorporated” built relationships with parents, religious leaders, and politicians to support youth SRH education (Action Health Incorporated, 2002), which played key roles in the acceptance of SRH education policy (UNESCO, 2018).

Gender norms also impact SRH policy implementation. A study in China found 25% of male students surveyed agreed that it is less important for girls to do well in school (UNESCO, 2018). In Indonesia, unmarried male adolescents do not face stigma if their female partner is pregnant, whilst unmarried, pregnant female adolescents face societal ostracization, are expelled from school, and are unable to access SRH services (Ngarari, 2010). Such beliefs can stem from family and community values and may deprioritize the sexual and educational needs of young females.

Promoting equality through education and advocacy can improve the school climate and access to SRH information (Amaro et al., 2001). Equity and fairness have been shown to positively impact SRH education policy implementation (Mathews et al., 2006), a trend which was also seen with education in other health domains (McCormick et al., 1995; Parcel et al., 2003). Furthermore, reducing stigma can improve the teaching environment, reducing teacher and student embarrassment during SRH education lessons (Rose et al., 2018), alongside improving class focus and relationships between teachers, students, parents, and the community.

One study looking at egalitarian values in countries in Africa, South America, and Asia found that increased levels of egalitarian values resulted in increased levels of condom use by adolescents, however, it was unclear whether this was more prevalent in females or not (Valverius et al., 2021). In such countries with high rates of adolescent pregnancies, it is important that these individuals, especially females experiencing adverse social effects as a result of pregnancy, feel empowered to make decisions about their sexual health.

#### Policy reform

The findings demonstrate a need to use student and teacher insight during SRH education policy formulation, to ensure policies align with the needs and realities of students (Samadaee Gelehkolaee et al., 2021). The importance of stakeholders in policymaking has been highlighted by several other articles, including how it benefits policy coherence and implementation (Lemke & Harris-Wai, 2015; OECD, 2021). Insight should include when lessons should be taught, the format of teaching, and creating adaptive implementation guidelines for varying school environments (Ponsford et al., 2018).

Involving stakeholders could have highlighted the issue of insufficient teacher training to policymakers, which was found to lead to insufficient policy implementation. Training was also shown to change teachers’ attitudes, making them more open to SRH education (Le Mat et al., 2021; Roodsaz, 2018).

Schools and teachers should be provided with policy documents, alongside tools and resources to aid implementation, especially as many teachers are expected to create their own syllabus. Furthermore, guidelines for regular monitoring seem necessary for evaluating implementation, holding organizations accountable, and identifying areas for improvement. Without monitoring systems, policies may fail to meet the demands of adolescents’ SRH needs, as seen In Vietnam, where new policies failed to meet targets due to a lack of an effective monitoring system (Khanh Chi et al., 2021).

#### Socioeconomic issues

Socioeconomic differences impact general education policy implementation, as highlighted in a study in South Africa (Raniga, 2014). In 2000, a national survey showed that 28% of schools in South Africa did not have access to water and sanitation (Human Science Research Council D of BE, 2000). Moreover, UNICEF data showed that around 29% of schools worldwide lacked access to basic drinking water and sanitation services (UNICEF DATA, 2023), a lack of which is detrimental to student educational outcomes. This is supported by current literature (Baker et al., 2002; Dundar et al., 2014; Frempong et al., 2011; Jasper et al., 2012; Johnson, 2008).

#### The need for comprehensive SRH education

The findings suggest a need to shift away from abstinence-only and fear-based education, which have been shown to be ineffective and withhold important information students need for healthy sexual relationships (Santelli et al., 2017). Teachers must set aside personal views when teaching SRH education, as adolescents are shown to be sexually active regardless of their opinions (Singh et al., 2019).

A different paper highlights the need for the inclusion of sexual pleasure in Comprehensive Sexuality Education (CSE), something which is explained to be vital for developing healthy sexual behaviors and empowering students (Mark et al., 2021). This, again, requires a shift in the cultural stigmas held by communities in these regions, so that students can have a well-rounded understanding of their sexual health.

One way to do this by using CSE itself to reduce stigma. For example, a study by Hong et al found that CSE reduced explicit and implicit stigma toward homosexual individuals (Hong et al., 2022). This evidence can be applied to reduce stigma in multiple contexts. One meta-analysis showed that, when compared to CSE, abstinence-only education did not reduce the likelihood of student pregnancy, and moreover could possibly increase it (DiCenso et al., 2002). Additionally, CSE is effective in reducing STI rates in low/middle-income countries, highlighting the need for its implementation in Asia and Africa (Fonner et al., 2014).

As marriage age is increasing (Statistics South Africa, 2021; Esteve et al., 2020; Greenspan, 1992), abstinence-until-marriage can be unrealistic for many, and a greater proportion of the population choose to have children later in life (Baryamutuma & Baingana, 2011; Remez et al., 2014). This makes the teaching of effective family planning all the more important (Kibret & Gebremedhin, 2022), as to empower individuals to control their life trajectory.

### Policy suggestions

1. It is important to involve key stakeholders in policy formulation to ensure better-informed SRH education policies
2. SRH education policy documents need to be supplemented with practical tools and guidance for implementation for schools and teachers
3. There is a need for consistent teacher training on SRH education and relevant policy implementation
4. Monitoring systems need to be developed to ensure gaps in implementation are identified and mitigated
5. School environments and cultures need to promote equity and sexuality rights
6. Socioeconomic issues^3^ in schools need addressing to allow comprehensive SRH education policy implementation

### Limitations and future research

The small number of included studies and the fact that these were mainly focused on Africa, means that the results may not be generalizable in other geographical areas with different health and education systems and cultures. Future research should focus on the factors shown to improve policy implementation in these regions, to assist the dissemination of learning from good practice.

## Conclusion

In conclusion, a small number of studies have been conducted on SRH education policy implementation in Asian and African schools.

Overall, the findings show inconsistent implementation of SRH policy in the Asian and African schools studied. Adolescent health seems to remain highly stigmatized. There is thus a need for a cultural shift and societal understanding of the importance of SRH, with policies adapted to facilitate uptake. Teachers′ training should sufficiently cover SRH education, and schools should be provided with the necessary tools and guidelines to ensure thorough curriculum implementation. Addressing basic school infrastructure needs, such as running water, would also assist toward the re-prioritization of comprehensive SRH education.

## Notes

1 Outside school hours.

2 Including teaching methods, values and topics taught.

3 Such as lack of general funding, access to water and sanitation, access to educational resources.

## Appendix

**Appendix A.** Keywords for literature search.

Themes/groups	Like/grouped terms
Adolescent	Adolescent OR Student OR School
Asia	Asia or Afghanistan OR Armenia OR Azerbaijan OR Bahrain OR Bangladesh OR Bhutan OR Brunei OR Cambodia OR China OR Cyprus OR Georgia OR India OR Indonesia OR Iran OR Iraq OR Israel OR Japan OR Jordan OR Kazakhstan OR Kuwait OR Kyrgyzstan OR Laos OR Lebanon OR Malaysia OR Maldives OR Mongolia OR Myanmar OR Nepal OR North Korea OR Oman OR Pakistan OR Philippines OR Qatar OR Saudi Arabia OR Singapore OR South Korea OR Sri Lanka OR Palestine OR Syria OR Tajikistan OR Thailand OR Timor-Leste OR Turkey OR Turkmenistan OR United Arab Emirates OR UAE OR Uzbekistan OR Vietnam OR Yemen
Africa	Africa OR Algeria OR Angola OR Benin OR Botswana OR Burkina Faso OR Burundi OR Cabo Verde OR Cameroon OR Central African Republic OR Chad OR Comoros OR Congo OR Cote d‘Ivoire OR Democratic Republic of Congo OR Guinea OR Eritrea OR Eswatini OR Ethiopia OR Gabon OR Gambia OR Ghana OR Kenya OR Lesotho OR Liberia OR Madagascar OR Malawi OR Mali OR Mauritania OR Mauritius OR Mozambique OR Namibia OR Niger OR Nigeria OR Rwanda OR Sao Tome and Principe OR Senegal Or Seychelles OR Sierra Leone OR South Africa OR South Sudan OR Togo OR Uganda OR Tanzania OR Zambia OR Zimbabwe
Policy	Policy OR Policies OR Curriculum
Sexual Health	Sexual Health OR SRH education OR Sex OR Reproductive Health OR Menstrual Health OR Menstruation
Education	Education

## Appendix

**Appendix B.** Database searches.

Database	Search
PubMed	(((Students[MeSH Terms] OR School[MeSH Terms] OR Adolescents[MeSH Terms]) AND (Afghanistan OR Armenia OR Azerbaijan OR Bahrain OR Bangladesh OR Bhutan OR Brunei OR Cambodia OR China OR Cyprus OR Georgia OR India OR Indonesia OR Iran OR Iraq OR Israel OR Japan OR Jordan OR Kazakhstan OR Kuwait OR Kyrgyzstan OR Laos OR Lebanon OR Malaysia OR Maldives OR Mongolia OR Myanmar OR Nepal OR North Korea OR Oman OR Pakistan OR Philippines OR Qatar OR Saudi Arabia OR Singapore OR South Korea OR Sri Lanka OR Palestine OR Syria OR Tajikistan OR Thailand OR Timor-Leste OR Turkey OR Turkmenistan OR United Arab Emirates OR UAE OR Uzbekistan OR Vietnam OR Yemen OR Asia OR Africa OR Algeria OR Angola OR Benin OR Botswana OR Burkina Faso OR Burundi OR Cabo Verde OR Cameroon OR Central African Republic OR Chad OR Comoros OR Congo OR Cote d‘Ivoire OR Democratic Republic of Congo OR Guinea OR Eritrea OR Eswatini OR Ethiopia OR Gabon OR Gambia OR Ghana OR Kenya OR Lesotho OR Liberia OR Madagascar OR Malawi OR Mali OR Mauritania OR Mauritius OR Mozambique OR Namibia OR Niger OR Nigeria OR Rwanda OR Sao Tome and Principe OR Senegal Or Seychelles OR sierra Leone OR South Africa OR South Sudan OR Togo OR Uganda OR Tanzania OR Zambia OR Zimbabawe)) AND (Policy[MeSH Terms] OR Policies[MeSH Terms] OR Law[MeSH Terms])) AND (Sexual[MeSH] OR Sexual Health[MeSH Terms] OR SRH education[MeSH Terms] OR Sex[MeSH Terms] OR Reproductive Health[MeSH Terms] OR Menstrual Health[MeSH Terms] OR Menstruation[MeSH Terms] OR HIV[MeSH Terms] OR AIDS[MeSH Terms])
Scopus	(TITLE-ABS-KEY ((sex OR hiv OR menstrual OR reproductive OR aids) AND education) AND TITLE-ABS-KEY (policy OR policies) AND TITLE-ABS-KEY (asia OR africa) AND TITLE-ABS-KEY (school OR adolescent OR student OR young)) AND PUBYEAR > 1999 AND PUBYEAR > 1999
Embase	(TITLE-ABS-KEY ((sex OR hiv OR menstrual OR reproductive OR aids) AND education) AND TITLE-ABS-KEY (policy OR policies) AND TITLE-ABS-KEY (asia OR africa) AND TITLE-ABS-KEY (school OR adolescent OR student OR young)) AND PUBYEAR > 1999 AND PUBYEAR > 1999
Web of Science	((((((AB=(Policy OR Policies OR curriculum)) AND AB=(School OR adolescent OR Student)) AND AB=(Sexual Health OR SRH education OR Sex OR Reproductive Health OR Menstrual Health OR Menstruation)) AND AB=(Education)) AND AB=(Afghanistan OR Armenia OR Azerbaijan OR Bahrain OR Bangladesh OR Bhutan OR Brunei OR Cambodia OR China OR Cyprus OR Georgia OR India OR Indonesia OR Iran OR Iraq OR Israel OR Japan OR Jordan OR Kazakhstan OR Kuwait OR Kyrgyzstan OR Laos OR Lebanon OR Malaysia OR Maldives OR Mongolia OR Myanmar OR Nepal OR North Korea OR Oman OR Pakistan OR Philippines OR Qatar OR Saudi Arabia OR Singapore OR South Korea OR Sri Lanka OR Palestine OR Syria OR Tajikistan OR Thailand OR Timor-Leste OR Turkey OR Turkmenistan OR United Arab Emirates OR UAE OR Uzbekistan OR Vietnam OR Yemen OR Algeria OR Angola OR Benin OR Botswana OR Burkina Faso OR Burundi OR Cabo Verde OR Cameroon OR Central African Republic OR Chad OR Comoros OR Congo OR Cote d‘Ivoire OR Democratic Republic of Congo OR Guinea OR Eritrea OR Eswatini OR Ethiopia OR Gabon OR Gambia OR Ghana OR Kenya OR Lesotho OR Liberia OR Madagascar OR Malawi OR Mali OR Mauritania OR Mauritius OR Mozambique OR Namibia OR Niger OR Nigeria OR Rwanda OR Sao Tome and Principe OR Senegal Or Seychelles OR sierra Leone OR South Africa OR South Sudan OR Togo OR Uganda OR Tanzania OR Zambia OR Zimbabwe OR Africa OR Asia)))
Global Index Medicus	Title, abstract, subject ((Policy OR Policies OR curriculum) AND (School OR adolescent OR Student) AND (Sexual Health OR SRH education OR Sex OR Reproductive Health OR Menstrual Health OR Menstruation OR HIV OR AIDS) AND (Africa OR Asia)) Title, abstract, subject (sexual health education policy)
Google Scholar	sex, health, polic*, education, adolescent, school Asia or Africa HIV, health, polic*, education, adolescent, school Asia or Africa menstrual, health, polic*, education, adolescent, school Asia or Africa menstrual, health, polic*, education, adolescent, school asia or africa sexual, health, polic*, education, adolescent, school asia or africa

## Appendix

**Appendix C.** PCC elements.

PCC Element	Definition
Population	Schools (Primary/Secondary) in Asia and Africa
Concept	Implementation of Sexual Health Education Policy, barriers in policy and implementation
Context	Articles published after 2000, nationally implemented policy, published in English

## Appendix

**Appendix D.** Included studies.

No.	Title	Authors	Year	Data collection	Data type	Focus	Country	Continent
1	An analysis of HIV/AIDS policy formulation & implementation structures, mechanisms & processes in the education sector in Kenya	Ndambuki et al.	2006	68 Questionnaires completed by teachers from 68 primary schools on implementation of policy	Mixed	Implementation, Barriers to implementation	Kenya	Africa
2	Factors associated with teachers’ implementation of HIV/AIDS education in secondary schools in Cape Town, South Africa	Mathews et al.	2006	324 questionnaires completed by teachers from 125 different schools	Mixed	Implementation, Barriers to implementation	South Africa	Africa
3	Protecting the Next Generation in Malawi: New Evidence on Adolescent Sexual and Reproductive Health Needs	Wittenberg et al.	2007	11 Focus group discussions with 14–19-year-olds (groups of 8–12), 102 in depth interviews with 12–19-year-olds (from secondary source)	Qualitative	Implementation, Barriers to implementation	Malawi	Africa
4	HIV education in South African schools: The dilemma and conflicts of educators	Ahmed et al.	2009	15 Interviews with principals and Grade 8 life-orientation educators	Qualitative	Barriers to implementation	South Africa	Africa
5	HIV/AIDS education in Kenya: an evaluation of policy, provision and practice in secondary schools	Ngarari	2010	447 questionnaires completed by students in various schools, 71 questionnaires from teachers from various schools, review of secondary data, policy analysis	Mixed	Implementation, Barriers to implementation	Kenya	Africa
6	Sexuality education: a ten-country review of school curricula in East and Southern Africa	Population Council (UNESCO and UNFPA)	2012	Review of curricula materials, review of secondary data	Qualitative	Policy, Implementation, Barriers to Implementation	Botswana, Kenya, Lesotho, Malawi, Namibia, South Africa, Swaziland, Uganda, Zambia, Zimbabwe	Africa
7	Challenges to providing HIV prevention education to youth with disabilities in South Africa	Rohleder et al.	2012	34 questionnaires completed by special education schools, 21 interviews with staff members	Qualitative	Implementation, Barriers to implementation	South Africa	Africa
8	Policy scripts and students’ realities regarding sexuality education in secondary schools in Kenya	Obare and Birungi	2013	Review of policy documents, 3624 questionnaires students aged 13–24 years (Secondary data)	Qualitative	Policy, Barriers to Implementation	Kenya	Africa
9	A critique of the South African National Life-skills and HIV/AIDS school policy: Lessons for policy adjustment	Raniga	2014	Review of policy documents, Structured interviews with principals from 74 secondary schools	Mixed	Policy, Implementation, Barriers to Implementation	South Africa	Africa
10	Teachers’ Understanding and Conceptualization of the HIV and AIDS Policy: The Case of Secondary Schools in Zimbabwe	Mugweni et al.	2014	Questionnaires, Interviews, Focus Groups with teachers from 4 secondary schools	Qualitative	Barriers to implementation	Zimbabwe	Africa
11	Sexuality Education in Malaysia: Perceived Issues and Barriers by Professionals	Khalaf et al.	2014	15 interviews with key professionals working in SRH in Malaysia	Qualitative	Barriers to implementation	Malaysia	Asia
12	Variable Implementation of Sexuality Education in Three Nigerian States.	Kunnuji et al.	2017	52 interviews with professionals with knowledge on the SRH curriculum	Qualitative	Implementation, Barriers to implementation	Nigeria	Africa
13	From Paper to Practice: Sexuality Education Policies and Their Implementation in Ghana	Awusabo-Asare et al.	2017	25 in depth interviews with key professionals in the field, questionnaires completed by teachers representing 81 schools and headteachers representing 78 schools, questionnaires completed by high school students aged 15–17 representing 81 schools	Mixed	Implementation, Barriers to implementation	Ghana	Africa
14	From Paper to Practice: Sexuality Education Policies and Their Implementation in Kenya	Sidze et al.	2017	25 in depth interviews with key professionals in the field, questionnaires completed by teachers representing 77 schools and headteachers representing 73 schools, questionnaires completed by high school students aged 15–17 representing 60 schools	Mixed	Implementation, Barriers to implementation	Kenya	Africa
15	Implementation of Sexuality Education in Middle Schools in China	UNESCO	2018	4737 student questionnaires, 151 teacher questionnaires, 29 headteacher questionnaires filled representing 30 schools, 70 in depth, semi-structured interviews with key stakeholders involved in SRH education	Mixed	Implementation, Barriers to implementation	China	Asia
16	Teachers’ views on the implementation of HIV/AIDS in schools: A case study of four high schools in the Fort Beaufort education district, Eastern Cape, South Africa	Koza and Mushoriwa	2018	12 semi-structured interviews with teachers, policy analysis	Qualitative	Implementation, Barriers to implementation	South Africa	Africa
17	Challenges to implementing national comprehensive sexuality education curricula in low- and middle-income countries: Case studies of Ghana, Kenya, Peru and Guatemala	Keogh et al.	2018	In depth interviews with 20/27 key informants, questionnaires conducted with 78/73 principals, 346/196 CSE teachers and 2990/2484 students aged 15–17 representing 82/78 secondary schools (Ghana/Kenya)	Mixed	Barriers to implementation	Ghana, Kenya, Peru, Guatemala	Africa, South America
18	Measuring the quality of sexuality education implementation at the school level in low- and middle-income countries	Keogh et al.	2019	346/196 structured interviews with teachers, 78/73 interviews with principals, 2990/2484 questionnaires completed by students (representing 82/78 schools) (Ghana/Kenya)	Mixed	Implementation, Barriers to implementation	Ghana, Kenya, Peru, Guatemala	Africa, South America
19	Moulding the teacher: factors shaping teacher enactment of comprehensive sexuality education policy in Ethiopia	Le Mat et al.	2019	56 interviews with teachers, parents, directors, young people, community leaders and governmental representatives	Qualitative	Barriers to Implementation	Ethiopia	Africa
20	Institutional and contextual obstacles to sexuality education policy implementation in Uganda	Ninsiima et al.	2019	Policy review, 32 in depth interviews and 4 focus group discussions with 32 key informants (teachers, district officials, NGO representative) on implementation, 8 in depth interviews with Ministry Officials, 4 religious leaders	Qualitative	Implementation, Barriers to implementation	Uganda	Africa
21	Barriers to sexual and reproductive education among in-school adolescents in Zomba and Mangochi districts, Malawi	Likupe et al.	2020	14 Focus Groups with 12 primary schools with 133 students between 10–19 years, 20 teachers and 25 parents	Qualitative	Barriers to implementation	Malawi	Africa
22	Conflicting HIV/AIDS SRH education Policies and Mixed Messaging among Educators and Students in the Lower Manya Krobo Municipality, Ghana	Ocran et al.	2022	31 interviews (20 students, 10 school-based health coordinaters, 1 Municipal Health Education Coordinator)	Qualitative	Barriers to implementation	Ghana	Africa
23	Challenges to ethical integration of reproductive health education in schools of Tshwane District, South Africa	Ramalepa et al.	2022	Questionnaires of 66 schools filled by principals and deputies	Mixed	Implementation, Barriers to implementation	South Africa	Africa
24	Unpacking the Contributing Factors of Inadequate SRH education at Schools in Bangladesh: Policy Recommendations	Roy, Hossain and Ghosh	2022	Literature review of secondary data	Qualitative	Barriers to implementation	Bangladesh	Asia
